# Formulation, implementation and evaluation of a distance course for accreditation in patient safety

**DOI:** 10.1590/S1679-45082018GS4316

**Published:** 2018-06-22

**Authors:** Poliana Nunes Wanderlei, Erik Montagna

**Affiliations:** 1 Faculdade de Medicina do ABC, Santo André, SP, Brazil.

**Keywords:** Patient safety, Education, distance, Education, continuing, Hospital accreditation, Educational measurement, Segurança do paciente, Educação a distância, Educação continuada, Acreditação hospitalar, Avaliação educacional

## Abstract

**Objective:**

To formulate and to implement a virtual learning environment course in patient safety, and to propose ways to estimate the impact of the course in patient safety outcomes.

**Methods:**

The course was part of an accreditation process and involved all employees of a public hospital in Brazil. The whole hospital staff was enrolled in the course. The accreditation team defined the syllabus. The education guidelines were divided into 12 modules related to quality, patient safety and required organizational practices. The assessment was performed at the end of each module through multiple-choice tests. The results were estimated according to occurrence of adverse events. Data were collected after the course, and employees’ attitude was surveyed.

**Results:**

More than 80% of participants reached up to 70% success on tests after the course; the event-reporting rate increased from 714 (16,264 patients) to 1,401 (10,180 patients).

**Conclusion:**

Virtual learning environment was a successful tool data. Data on course evaluation is consistent with increase in identification and reporting of adverse events. Although the report increment is not positive *per si*, it indicates changes in patient safety culture.

## RESUMO

**Objetivo:**

Elaborar e implantar um curso de segurança do paciente em um ambiente virtual de aprendizagem, e propor métodos para estimar seu impacto nos resultados de segurança do paciente.

**Métodos:**

O curso fez parte de um processo de acreditação e envolveu todos os colaboradores de um hospital público. Toda a equipe do hospital foi matriculada no curso. A equipe de acreditação definiu o programa. As diretrizes de instrução foram divididas em 12 módulos relacionados à qualidade, segurança do paciente e práticas organizacionais requeridas. A avaliação foi realizada no final de cada módulo, por meio de testes de escolha múltipla. Os resultados foram estimados pela ocorrência de eventos adversos. Os dados foram coletados após o curso, e a atitude dos empregados foi pesquisada.

**Resultados:**

Mais de 80% dos participantes atingiram até 70% de sucesso em testes; após o curso, a taxa de notificação de eventos aumentou de 714 (16.264 pacientes) para 1.401 notificações (10.180 pacientes).

**Conclusão:**

O ambiente virtual de aprendizagem foi uma ferramenta bem-sucedida. Os dados da avaliação do curso são coerentes com o aumento da identificação e da notificação de eventos adversos. Embora o incremento de notificação não seja positivo por si só, é indicativo de mudanças na cultura de segurança do paciente.

## INTRODUCTION

In 1999, the publication of the report entitled To Err is Human: Building a Safer Health System, by the United States Institute of Medicine (IOM) was a historical milestone. It warned about estimated deaths from adverse events related to patient care, which rose from 44 thousand to 98 thousand cases in 1 year. Deaths caused by healthcare-related errors were classified as the eighth cause of mortality in the United States.^(^ ^1^ ^)^

Ten years after, the Lucian Leape Institute, at the National Patient Safety Foundation, launched a study showing deficiencies in medical student training, from basic knowledge to skills required to provide safe patient’s care. This is the first of a series of studies that identified priorities in organizing courses to improve patient safety.^(^ ^2^ ^)^

The need for educating healthcare professionals on patient safety has been established and is recognized as a fundamental part of their training. Moreover, it is important to emphasize the compulsory aspect of training on patient safety to all hospital employees. Medical residency programs should also include quality of care for trainees, as required by the Accreditation Council for Graduate Medical Education (ACGME).^(^ ^3^ ^)^

However, the reach of education programs is limited by several factors within a healthcare institution: the variety of professions, professional performance, hiring of lecturers, classrooms, didactic material, as well as suitable allocation of time and space required for classes. Likewise, other limiting factors include costs of programs and the necessary time to promote personnel training.

Thus, online education emerges as an alternative that has some advantages, such as no need for a physical space (classrooms, *e.g*.); can be offered simultaneously to all employees (which decreases the total training time, with no the need to set up a group); it allows employees to adjust the training to their routine, not interfering in their work schedule; allows students to have access to their learning environment according to their convenience; and higher student/lecturer ratio, depending on the strategy. These factors are relevant in scenarios with limited financial resources. That is the case in the present study, aiming to meet the specific needs and limitations of students and organizations.^(^ ^4^ ^)^

## OBJECTIVE

To prepare and implement a virtual learning environment course in patient safety at a hospital, besides putting forward ways to estimate the impact of the course on patient safety outcomes.

## METHODS

This is a descriptive, exploratory, cross-sectional field study, with a quantitative and qualitative approach carried out in 2014. It was based on data collection on performance of the participants and organizations during the accreditation process, as well as on patient safety indicators. Ethical issues were considered based on Lynn et al.,^(^ ^5^ ^)^ and the data was obtained in compliance with current policy on ethics review.^(^ ^6^ ^)^

This study was carried out at a public state hospital, which characteristically had severe asset limitations, high turnover of employees, and a demand for services above the World Health Organization (WHO) recommendations.^(^ ^7^ ^)^ As agreed by the State Health Authority, the hospital was administered by a non-for-profit organization or a foundation, which complied with the requirements of State Law 2.675/2011, being regulated and qualified as a Healthcare Social Organization. The hospital had 1,535 professionals in the period of this study.

The course was designed to prepare the staff for the Accreditation Program, and all employees were expected to take part.^(^ ^8^ ^)^ The training program through a virtual learning environment (VLE) for an accreditation process was not known at the organization.

In October 2007, the hospital was accredited by the National Accreditation Organization (ONA - *Organização Nacional de Acreditação*). In 2010, it was Fully Accredited, and Accredited with Excellence, in 2011. By the end of 2011, when this study was initiated, the hospital applied to the Accreditation Canada program assessment, which guides and monitors high performance standards in quality and safety, with international criteria and global validation.

The study was approved by the Research Ethics Committee, under the resolution 1.333.258, CAAE: 50072015.0.0000.0083.

### Personnel profile

The target audience comprised healthcare professionals, technical and administrative staff. They were divided according to their occupation in the organization to monitor work as type 1, if operation staff (66%); type 2, staff with intermediate leadership (14%); and type 3, coordination and supervision staff (20%).

It is important to emphasize that due to low salaries, it is common for healthcare professionals to have more than one job, therefore, long working hours. In addition, their working routine is hampered by for living in a city with over 12 million inhabitants, which has problems such as stress, urban mobility, long commuting distances, high living costs and work overload.^(^ ^9^ ^)^

### Accreditation program

The Canadian Council on Health Services Accreditation (CCHSA) assists establishing and improving healthcare accreditation systems. The program aims at quality of patient care, administrative management and productivity optimization. Thus, activities and services are pre-established through the creation of protocols and patient care flows, focusing on the principles of excellence aligned with patient safety.^(^ ^10^ ^)^

The Accreditation Manual follows a management model that assesses the institutional policy of patient safety, emphasizing sustained improvements in quality and safety, particularly in patient safety. To this end, the accreditation process should involve the identification of conditions and practices, highlight and correct those that are hazardous to the patient, encourage more reporting/notifications, and reinforce the capacity to reduce risks, thus contributing to a continuous improvement of quality.^(^ ^10^ ^)^ The organizations pursuing the certification should apply voluntarily and prepare for a survey.

### Course structure and design

The phases of the course comprise: VLE platform customization and preparation of course content in modules; implementation and presentation; enrolment and evaluation of learning.

#### First phase: platform customization and preparation of course content in modules

Moodle was the learning platform chosen for the VLE, primarily because it is a free and dynamic open source platform. These characteristics result in low cost and many possibilities of customization. In addition, its programming language and database provide stability that does not require a dedicated local server, allowing communication with various operating systems with no compatibility conflicts.^(^ ^11^ ^)^ Furthermore, its tools offer educational material that help with questionnaire formulation through the aid of a content database.

Study progression was based on the reading materials provided and on assessment questions that could be answered at any time. At the end of each module, the students had to answer all questions and they would pass to the next module only after completing the task. Questions were answered with no interaction with tutors. All employees were given 3 months to complete the course.

Course syllabus, content set up, as well as evaluations, were based on the subjects that are part of the Accreditation Canada Manual, and are required for training programs of the organizations that apply for this accreditation. The syllabus was defined by the accreditation board guidelines^(^ ^10^ ^)^ and comprised the following topics:

- Module I – Quality: introduction and description of the Accreditation Canada program.
- Module II – Patient safety: definitions, terms, guidelines, and explanation about patient safety; practices the organization should use; introduction to the Required Organizational Practices (ROPs) to promote patient safety.
- Module III – ROPs: definitions of all ROPs submitted to the Accreditation Canada program. ROPs are checkpoints performed for a safer patient care.^(^ ^10^ ^)^ They were divided into five different areas: communication; use of medication; working environment; hospital infection control; and creation of a culture of safety in the organization.

#### Second phase: implementation and course presentation

Users were enrolled in the VLE course by the Human Resources Department and received a login name and a password through the intranet. They also received an user’s guide developed for the course, orienting the student’s work through a relevant helpdesk.

#### Third phase: enrolment and learning evaluation

The Human Resources Department monitored employees’ enrolment and grades through an administrator login. Access verification of participants were collected through a navigation log provided by the VLE, as well as the following information: which modules were accessed, which materials were visited, how many students completed the proposed tasks and performed the assessment, and finally, the student’s performance in the evaluation.

An assessment was carried out at the end of each module completed by the employee. Grades were generated by the system and the access time to the platform by the participant was also recorded. Those who scored below 75 out of 100 points had to take the module once again. Whenever was the case, a VLE’s random question assignment provided very low chance of task repetition for the same participant.

#### Fourth phase: personnel survey on patient safety culture

Outcomes in patient safety culture among participants were held by a Likert-type survey, the Hospital Survey on Patient Safety Culture (HSOPSC). This survey was proposed by the Agency for Healthcare Research and Quality (AHRQ), and assesses personnel attitude changes on patient safety culture through a multidimensional approach.^(^ ^12^ ^)^ The validity of the test was confirmed by Cronbach’s α.^(^ ^13^ ^)^ Results were obtained after the accreditation process to estimate the maintenance or adhesion to the process, being compared in 2016 and 2017. Data from staff performance and risk stratification are presented in terms of absolute and relative frequency.

## RESULTS

### Overall staff performance

**Figure 1:**
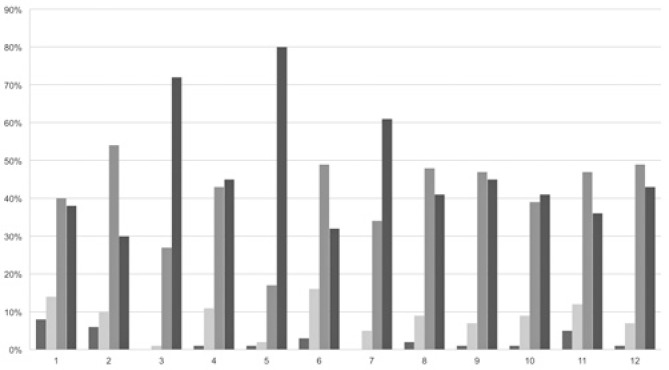
Overall performance of type 1 employees

**Figure 2:**
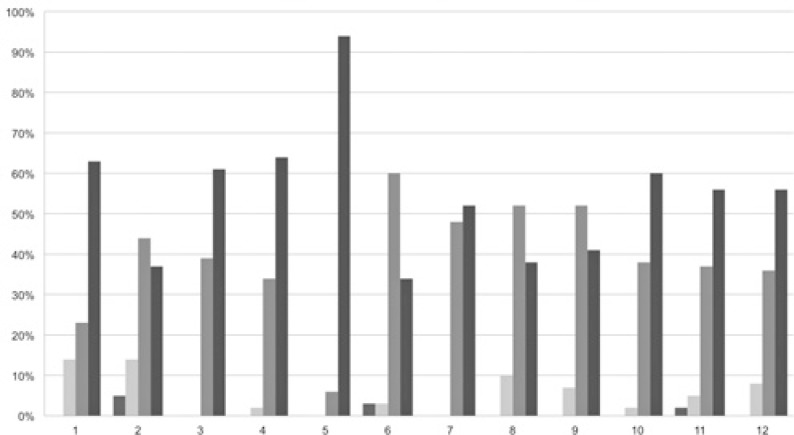
Overall performance of type 2 employees

**Figure 3:**
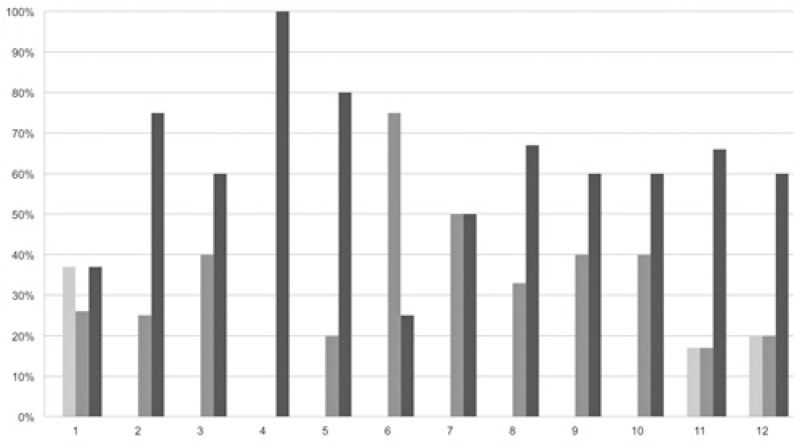
Overall performance of type 3 employees

Out of the 1,535 employees of the organization, 859 attended the patient safety course and answered the final questions. Those who attended the course, 98% passed and 2% failed. It is presented the percentual occurrence of grades (y axis) by type 1 employees along the course modules (x axis). Each group of four columns is related to the possible grades, from left to right − zero, 3, 7 and 10. The performance for each type of occupation on each course module is displayed on figures 1 to 3.

### Impact of the course on the organization

After the patient safety course, data was collected to assess its impact on the organization.

To refine data analysis, notifications were created to identify the occurrence of adverse events, that is, notifications show the unexpected or unwanted results that affect patient safety. Some examples of adverse events include patient falls or risk of falling, patient misidentification, medical examination switch, failure to administer a medication, surgical procedure failure, bedsore development, infections, accidental extubation, patient injury, obstetric trauma, among others. Thus, events that potentially have harmful consequences to the patient should be reported. Employees which incorporate ROPs to their daily practice are more likely to avoid or minimize adverse events.^(^ ^14^ ^)^

The number of adverse event reports evolved in the period 2014-2016 as follows: in 2014, there were 457 reports for 15,444 (2.96%) hospitalized patients. After the course, this number increased to 714 for 16,264 patients (4.39%) and, in the first semester of 2016, it went up to 1,401 notifications out of 10,180 patients (13.76%).

**Figure 4:**
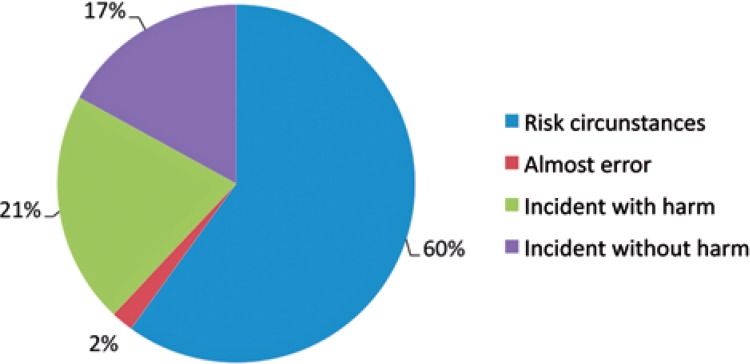
Risk stratification in the first semester of 2016

The WHO International Classification for Patient Safety (ICPS) deals with risk circumstances, which include identification of risks, circumstances and situations that have not yet caused harm to patients and staff.^(^ ^14^ ^)^ The following data depicted in figure 4 show the individual perception of staff regarding risks in the period, which helps improving investigation, identification, reduction and/or elimination of risk circumstances as well as their associated factors.

### Staff survey on patient safety culture

Tables 1 to 3 represent the HSOPSC. The consistence of responses was measured by the Cronbach’s α.

**Table 1:** Seven dimensions related to hospital departments or working units

Dimensions		2016	2017
Communication openness, %			
	Positive	76	73
	Negative	24	27
		α=0.79	α=0.84
Feedback and communication about error, %			
	Positive	82	83
	Negative	18	17
		α=0.81	α=0.88
Organizational learning – continuous improvement, %			
	Positive	77	75
	Negative	12	13
	Neutral	11	12
		α=0.77	α=0.83
Supervisor/manager expectations & actions promoting patient safety, %			
	Positive	61	62
	Negative	24	23
	Neutral	15	15
		α=0.80	α=0.81
Non-punitive response to errors, %			
	Positive	35	35
	Negative	49	49
	Neutral	16	16
		α=0.81	α=0.93
Teamwork within units, %			
	Positive	71	71
	Negative	20	19
	Neutral	9	10
		α=0.72	α=0.73
Staffing, %			
	Positive	36	39
	Negative	47	45
	Neutral	17	16
		α=0.74	α=0.77

**Table 2:** Three dimensions to assess safety culture awareness at hospital

Dimensions		2016	2017
Management support for patient safety, %			
	Positive	70	68
	Negative	16	17
	Neutral	14	15
		α=0.81	α=0.80
Handoffs & transition, %			
	Positive	47	45
	Negative	29	32
	Neutral	24	23
		α=0.70	α=0.72
Teamwork across units, %			
	Positive	60	56
	Negative	21	23
	Neutral	19	21
		α=0.77	α=0.80

**Table 3:** Two dimensions to evaluate results

Dimensions	2016	2017
Overall perceptions of patient safety, %		
Positive	61	61
Negative	24	24
Neutral	15	15
	α=0.87	α=0.82
Frequency of events reported, %		
Positive	84	83
Negative	16	17
	α=0.94	α=0.96

## DISCUSSION

### Overall staff performance

The high success rate suggests that adopting a VLE was a feasible approach. The VLE allowed employees to have remote access to the course. The flexible schedules to perform their tasks according to their convenience and routine may have contributed to accomplishing tasks and may have been a significant factor for the overall performance achieved.

It should be noted that none of the training programs or courses offered by the Human Resources Department has ever been carried out using distance learning. Moreover, addressing the same subject repeatedly to different audiences, in a short period, would be more demanding from the instructor’s point of view.

The choice for an open source VLE involved almost no financial cost to the institution, besides the demand for operational computing structure, which is already available. Actually, a low impact on budget was observed, since the whole process only involved the reallocation of available human resources. From the perspective of a public health organization in a country with serious budgetary constraints, low cost is a relevant factor. We suggest that the organization involvement is crucial for success of the course. This support can be demonstrated by the low rate of absence and dropouts.

A remarkable aspect of the chosen design of the course is the challenge to train and evaluate professionals with distinct occupations, levels of education, and hierarchical positions in the organization and, above all, logistical, structural as well as operational constraints.

Although there are data on training programs, they are primarily related to interns, residents and healthcare professionals,^(^ ^15^ ^-^ ^17^ ^)^ not to other professionals. Successful experiences have been reported about medical and health sciences undergraduate students in Brazil,^(^ ^18^ ^,^ ^19^ ^)^ as well as didactic approaches for similar attendances.^(^ ^20^ ^,^ ^21^ ^)^ There are also some differences between the structure of courses held at teaching hospitals^(^ ^22^ ^,^ ^23^ ^)^ and the data herein presented.

It is arguable if the difficulty level of questions was low. However, this data cannot be analyzed alone and we must take into account the impact on the organization.

### Impact of the course on the organization

Reporting is the main tool in risk management, since it permanently improves intervention processes, which is the key to successful certification programs.^(^ ^24^ ^)^ Indeed, the first indication of effectiveness of the course was the comparison of total number of event notifications related to direct or indirect patient care, carried out by the risk management sector, in 2014, 2015 and the first semester of 2016. The comparison was made in all areas by means of data obtained before and after the course presentation. The results herein presented were comparable to those of similar organizations.^(^ ^25^ ^)^

The evolution of adverse event reports in 2014 to 2016 suggests an increasing identification and reporting by employees. Although the increased number of notifications is not positive *per si*, it is licit to suppose that employees were more prepared to identify the events after the course. This data contributes to including more reports by other professionals,^(^ ^26^ ^)^ as shown by staff perception. It is an important step towards continuous improvement of procedures, and it should be followed up for quality control and data collection for further studies and interventions.

The perception about patient safety level (HSOPSC) among employees was generally positive. The results of working units showed a positive perception (>75%) regarding communication and reporting (items 1 to 3). This data suggests that the increased number of reports presented should be an expected consequence of the course. Items 4 to 7 showed that management personnel could be perceived as punitive, although teamwork among units is over 70%. This data reveals the need to enhance leadership attitudes and staff response to hierarchy. However, previous reports discuss data constraints on staff response in Brazilian hospital culture,^(^ ^23^ ^,^ ^25^ ^)^ and results may differ significantly.

Results of higher hierarchy professionals showed lower integration as compared to unit staff. It reinforces the need to improve leadership and raises questions whether the course effects are more positive on lower staff levels.

The degree of patient safety in the organization was evaluated as positive by 61% of participants. These data are considered of concern,^(^ ^21^ ^)^ although improvement was observed from an year to another. Furthermore, previous data points there are few studies on patient safety culture, mainly from developed countries.^(^ ^27^ ^)^ The studies available on low-income countries or those with marked social inequities showed low scores.^(^ ^28^ ^-^ ^30^ ^)^

Finally, despite the very similar results from 2016 to 2017 on patient safety culture survey, the greater Cronbach’s α coefficent suggests a more consistent answer by employees. This parameter considers the internal validity of the survey and should be above 0.70. It means stronger results than the year before, which along the main outcomes, may reveal that the staff – who performed procedures not identifying possible problems – changed to a better attitude.

## CONCLUSION

The patient safety course played a central role in the accreditation and certification process, as was decisive to obtain the quality seal. In addition, knowledge acquired contributed to understanding the needs to systematically adopt the required organizational practices, and to enhancing patient safety culture. Regardless of legal requirements to implement patient safety programs, its execution requires willingness of managers and collaboration by all those involved in the process.

The most relevant outcome of this study was the increased number of event reports in the organization, suggesting the training program was probably successful, and improved patient safety culture. The survey also detected support of the staff to the accreditation process, thus reflecting on an improved patient safety culture.
